# Dataset from the life cycle assessment and techno-economic analysis of net and longline nearshore *Saccharina latissima* cultivation systems

**DOI:** 10.1016/j.dib.2026.112742

**Published:** 2026-04-04

**Authors:** Arrate Sainz de la Maza Larrea, Manali Chakraborty, Teis Boderskov, Mette Møller Nielsen, Annette Bruhn, Marianne Thomsen

**Affiliations:** a Copenhagen University, Department of FOOD, Rolighedsvej 26, 1958 Frederiksberg, Denmark; b Aarhus University, Department of Ecoscience, C.F. Møllers Allé 1110, 1120, 1130 & 1131 8000 Aarhus, Denmark; c Aarhus University, Centre for Circular Bioeconomy (CBIO), Blichers Allé 20, 8830 Foulum, Denmark; d Technical University of Denmark, National Institute of Aquatic Resources, Øroddevej 80 7900 Nykøbing Mors, Denmark

**Keywords:** Life cycle inventory (LCI), Net present value (NPV), Seaweed cultivation, Carbon footprint, Biomass yield, Marine aquaculture, Coastal restoration

## Abstract

Seaweed cultivation is considered a potential tool to face the environmental pressures derived from the intensification of global food demand. This article presents a dataset supporting a comparative sustainability assessment of two nearshore cultivation systems, longline and tube-net, for *Saccharina latissima* in Danish waters. In this work, a comprehensive Life Cycle Assessment (LCA) was used to quantify the environmental performance of two main nearshore cultivation systems: traditional longline and tube-net setup. The system boundary includes the whole life cycle of the seaweed production until the harvest, excluding the distribution and end-of-life stages. The dataset is based on empirical pilot-scale data collected at two cultivation sites in Limfjorden (Denmark) and scaled to a reference farm area of 18.75 ha. Foreground data were obtained from pilot cultivation sites located in Limfjorden (Denmark), while background data were sources from Ecoinvent 3.10 and AGRIBALYSE v3.1.1. Environmental impacts are presented for several categories included in the ReCiPe (H) 2016 method, covering a wide range of environmental impacts. Results are provided for multiple functional units, including per cultivation site per year, per hectare, per kg fresh biomass, per kg dry biomass, and per meter of cultivation infrastructure.

Moreover, a Techno-Economic Assessment (TEA) with a 7% discount rate and several seaweed prices is included, evaluated over a 10-year project horizon using Net Present Value (NPV). The dataset includes detailed Life Cycle Inventories (LCI), Life Cycle Impact Assessment (LCIA) results, techno-economic calculations, and modelling assumptions, enabling transparency, reproducibility, and reuse in future environmental and economic assessments of seaweed cultivation systems.

Specifications Table

**Table:** 

Subject	Earth & Environmental Sciences
Specific subject area	Environmental and techno-economic assessment of *Saccharina latissima* cultivation based on different production systems
Type of data	Table, figure and equation Raw and analysed data
Data collection	Inventory data were collected from the pilot seaweed farm and extrapolated to a full-scale farm of 18,75 ha. In the absence of primary data, secondary data were obtained from secondary sources, including scientific and technical literature. Background data mainly comes from the database Ecoinvent 3.10 and AGRIBALYSE v3.1.1. Life Cycle Impact Assessments were performed using SimaPro v9.1 software and the method “ReCiPe 2016 Midpoint (H) method“.
Data source location	Limfjorden, Denmark Coordinates: 56° 33.046′ N 08° 35.232′ E (for net system) and 56° 47.093′ N 08° 54.913′ E (for longline system)
Data accessibility	Repository name: Zenodo Data identification number: 10.5281/zenodo.18016121 Direct URL to data: https://zenodo.org/uploads/18016121
Related research article	Chakraborty M., Sainz de la Maza Larrea, A., Boderskov T., Nielsen, M. M., Bruhn A., Thomsen M. Comparative Sustainability Assessment of Net and Longline Nearshore Cultivation Systems for *Saccharina latissima* in Danish Waters. Algal Research. In Review [1]

## 1 Value of the Data

• This data represents the Life Cycle Inventories (LCI) and Life Cycle Impact Assessments (LCIA) of different *Saccharina latissima* production systems in Denmark.
• The LCA and LCIA described in this article provide methodological details and ensure transparency in the LCA model of the related research article.
• The detailed inventory LCA methodology can serve as a base for future research on this topic.
• The rigorous environmental data is combined with a net present value calculation that serves as a benchmark for the economic viability of seaweed cultivation.

## 2 Background

Seaweed’s multifunctional benefits have turned it into a key player in the blue economy. Seaweed such as *Saccharina latissima*, can contribute to climate resilience through its ecosystem services. The assimilation of carbon and dissolved inorganic nutrients supports nutrient cycling and marine habitat restoration [2,3]. Thus, seaweed cultivation can be very valuable for sustainable resource management and marine bioremediation. Globally, several offshore seaweed cultivation systems have already been successfully implemented. Some of these systems have been studied in detail from an environmental and economic perspective, and relevant studies have provided comprehensive inventories and descriptions of the product systems [[4], [5], [6], [7]]. The dataset presented in this work focuses on a novel seaweed cultivation system, tube-net, which has been adapted from mussel production. This work supports the results and conclusions of the original research article that compares the novel cultivation system with a traditional system. By providing detailed Life Cycle Inventories, Life Cycle Impact Assessments and Net Present Value calculations, this work contributes to: (i) determining the environmental hotspots of different seaweed cultivation systems; (ii) comparing the environmental performance of those systems; and (iii) evaluating their financial viability.

This Data in Brief article serves as the primary source of the complete dataset underlying the associated research article in Algal Research [1]. While the associated research article presents aggregated results and their interpretation, the present article provides the full life cycle inventories, modelling assumptions, intermediate calculations, and detailed LCA and TEA outputs required for transparency, reproducibility, and data reuse. The supplementary material of the associated research article contains only aggregated results supporting its figures and tables and does not duplicate the full dataset reported here.

## 3 Data Description

This article contains data related to the inventory data of 4 different nearshore production systems of *Saccharina latissima* and their respective LCIA and TEA. The dataset presented in this article constitutes the complete and detailed documentation of the environmental and techno-economic assessment, including full inventory data, modelling assumptions, and calculation procedures. It is intended to complement the associated research article by providing the full methodological transparency and reproducible data that are not included there. Data includes information on the operation and construction stages, such as materials, infrastructure and manpower. The attached Excel file contains the following information:

1. Biomass composition: carbon, nitrogen and phosphorus composition of the seaweed biomass harvested on each pilot cultivation setup, together with the fresh and dry weight. Numbers are given in percentage and absolute numbers.
2. Net setup _inventory: Life Cycle Inventory for the different seaweed production systems that use a net setup. The main difference between them is the weight system used to hold the net. The inputs to the inventory are divided into hatchery and cultivation phases, which are subdivided into construction and operation stages. The dataset contains the following information about each input: amount per shipping container and/or cultivation site, lifetime and price per unit. The file also contains farm data such as seaweed biomass yield, cultivation area and meters of seeded rope.
3. Line setup_inventory: Life Cycle Inventory for the seaweed production system that uses a line setup. The inputs to the inventory are divided into hatchery and cultivation phases, which are subdivided into construction and operation stages. The dataset contains the following information about each input: amount per shipping container and/or cultivation site, lifetime and price per unit. The file also contains farm data such as seaweed biomass yield, cultivation area and meters of seeded rope.
4. LCI_net setup: detailed Life Cycle Inventory which includes disaggregation of inputs in the “net setup_inventory” into single items; e.g. a specific material, diesel for boat operation, or a production or transformation process. The sheet also contains the background unit processes used for each item and the assumptions made for the calculations.
5. LCI_line setup: detailed Life Cycle Inventory which includes disaggregation of inputs in the “line setup_inventory” into single items; e.g. a specific material, diesel for boat operation, or a production or transformation process. The sheet also contains the background unit processes used for each item and the assumptions made for the calculations.
6. LCIA_net setup: Life Cycle Impact Assessment results obtained for the yearly production of *S. latissima* in a cultivation site of 18,75 ha when using different net setup systems. The environmental indicators analysed are those included in the ReCiPe 2016 Midpoint (H) method: fine particulate matter formation, fossil resource scarcity, freshwater ecotoxicity, freshwater eutrophication, global warming, human carcinogenic toxicity, human non-carcinogenic toxicity, ionizing radiation, land use, marine ecotoxicity, marine eutrophication, mineral resource scarcity, ozone formation (human health), ozone formation (terrestrial ecosystems), stratospheric ozone depletion, terrestrial acidification, terrestrial ecotoxicity and water consumption. Results are given per each inventory item and as well as aggregated into a total impact score per cultivation site.
7. LCIA_line setup: Life Cycle Impact Assessment results obtained for the yearly production of *S. latissima* in a cultivation site of 18,75 ha when using a line setup system. The environmental indicators analysed are those included in the ReCiPe 2016 Midpoint (H) method: fine particulate matter formation, fossil resource scarcity, freshwater ecotoxicity, freshwater eutrophication, global warming, human carcinogenic toxicity, human non-carcinogenic toxicity, ionizing radiation, land use, marine ecotoxicity, marine eutrophication, mineral resource scarcity, ozone formation (human health), ozone formation (terrestrial ecosystems), stratospheric ozone depletion, terrestrial acidification, terrestrial ecotoxicity and water consumption. Results are given per each inventory item and as well as aggregated into a total impact score per cultivation site.
8. LCIA (FU comparison): Life Cycle Impact Assessment results for all the production systems given per different functional units: cultivation area, hectare, kg of fresh biomass, kg of dry biomass, and meters of pipe or mainline. Data in “LCIA_net” and “LCIA_line” are transformed using data from “Net setup_inventory” and “Line setup_inventory”.
9. Contribution analysis: Compiled LCIA results for the three net setups and the longline system using the full cultivation area as the functional unit. Net environmental impact is also included after adjusting the impacts for the assimilated emissions.
10. Groupped contribution: Compiled LCIA results for the three net setups and the longline system. The single items in the LCI and contribution analysis are grouped into thirteen main categories for a better visual representation. Results are given per cultivation site.
11. Sensitivity analysis: Life Cycle Impact Assessment results for the four different sensitivity scenarios: lighter pipe (110 nm), 5-year lifetime of net and longline, a combination of a longer lifetime and a lighter pipe, and Ecoinvent’s shipping container. Results are given per cultivation site.
12. NPV_Net: Detailed analysis of techno-economic assessment of the net setup includes net profit value (NPV) of the systems under varying end-use market prices and a 7% discount rate over a period of 10 years for the full cultivation area. Results are given in €.
13. NPV_Line: Detailed analysis of techno-economic assessment of the Line setup includes net profit value (NPV) of the systems under varying end-use market prices and a 7% discount rate over a period of 10 years for the full cultivation area. Results are given in €.
14. F2_medium: composition of f/2 medium used in the hatchery phase.
15. This dataset represents the sole source of full inventory-level data, modelling assumptions, and calculation procedures supporting the associated research article, enabling independent verification and reuse.

## 4 Experimental Design, Materials and Methods

### 4.1 Seaweed production

**Fig. 1:**
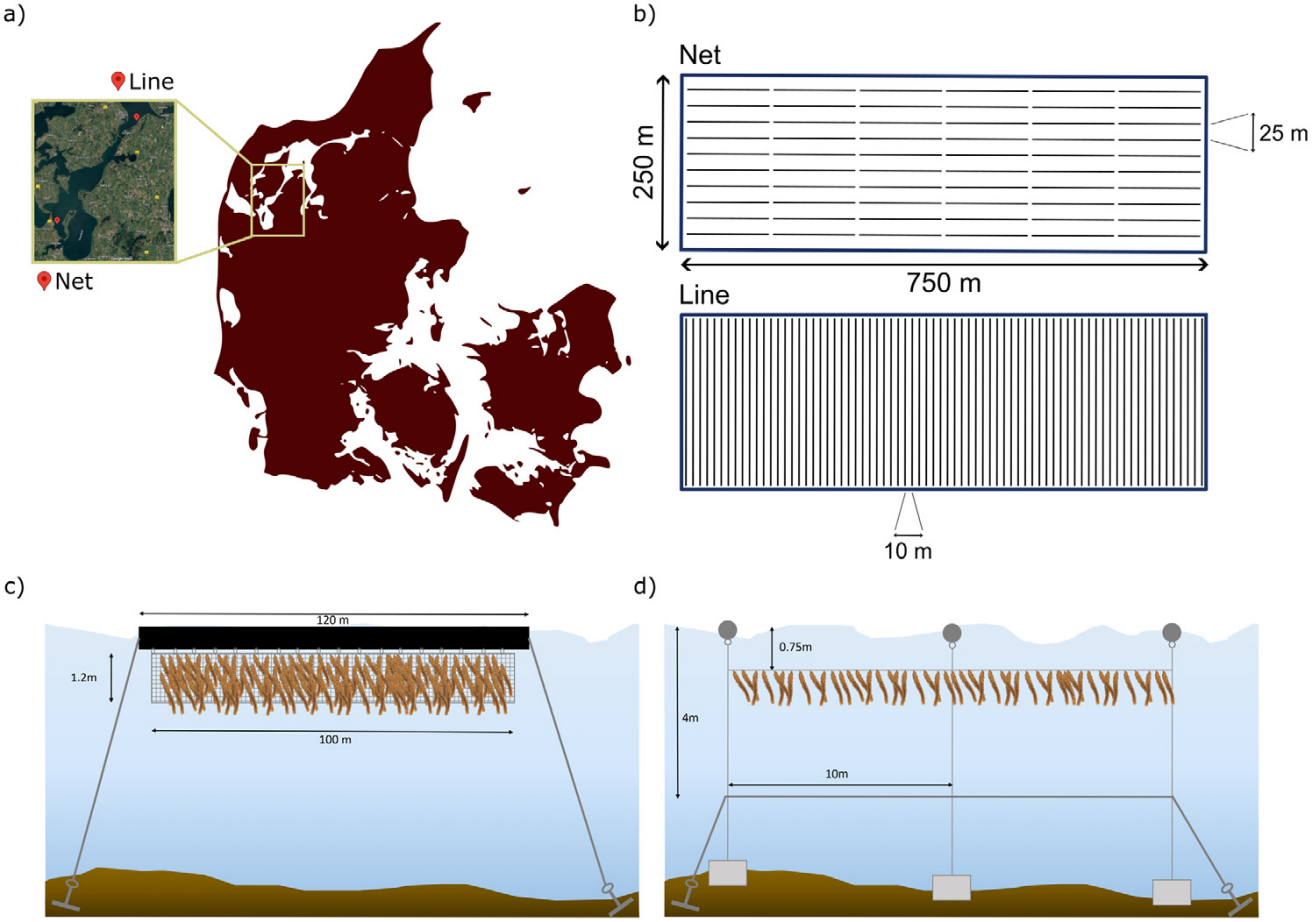
Net and line nearshore cultivation systems. a) Geographical coordinates of the pilot farm (N 56° 33.046′ E 08° 35.232′ for the net system, and E 08° 54.913′ N 56° 47.093′ for the line). b) Simulated bird’s eye view of the full-scale (18.75 ha) farm with a distance between the pipes and mainlines of 25 m and 10 m in the net and line setups, respectively. c) Schematic representation of the net system. d) Schematic representation of the line system. The figure was taken from the associated manuscript [1].

This study is based on two separate seaweed grow-out pilots conducted at two commercial coastal farms in the Limfjorden, Denmark (Fig. 1a). The trials tested two distinct cultivation systems—a line-based setup and a net-based setup—each with different farm configurations and infrastructure (Fig. 1b-d). Both trials used the same hatchery setup for initial seeding. For simplicity, longline and tube-net setups will be refer to as line and net setups.

### 4.2 Hatchery

Seaweed seeding was carried out in a mobile hatchery unit housed in a 40-foot cooling container, equipped with 18 tanks. The total seeding capacity is 900 m of net or 21,600 m of line per container. Nets were placed directly into the tanks, while lines were coiled onto PVC pipes for easier handling. Each tank was filled with filtered (0.2 µm) and UV-treated seawater, and equipped with a circulation pump, two air stones, and an overhead light source to ensure adequate water movement, gas exchange, and light for seaweed growth. Seeding was performed using a spore solution (3500 spores/mL), followed by a 7-week hatchery phase. During this period, nutrients were added to support growth, and germanium dioxide (GeO₂) was used to suppress diatom development. After 7–10 weeks, the seeded nets and lines were transferred to the farm for cultivation.

### 4.3 Cultivation

#### 4.3.1 Longline setup

The line-based cultivation trial was conducted within a 3-hectare section of the farm near Nykøbing Mors. Seven longlines, each 150 m in length, were installed at a depth of 4–6 m. Concrete blocks were installed for every 5 m to lower the mainline, and buoys were placed every 10 m as support for the seaweed lines, which were positioned horizontally above the mainline at a depth of approximately 0.75 m, ensuring optimal exposure to light and water flow (Fig. 1d).

No external inputs, such as nutrients or chemicals, were added during the farm phase; seaweed growth relied entirely on natural seawater conditions.

#### 4.3.2 Tube-net setup

The net-based cultivation trial used infrastructure adapted from mussel farming, with a tube-and-net configuration. Each unit consisted of a 120-meter-long tube (diameter: 315 mm) that provided buoyancy. From each tube, two 50-meter-long nets with a vertical depth of 1.2 m were suspended (Fig. 1c). The trial was conducted using two tubes, corresponding to 4 nets or 200 m of cultivation net in total.

To stabilise the nets in the water column, three different weighting methods were tested: i) concrete blocks, 1 L concrete weights spaced and attached with line every 1.2 m along the net; ii) heavy chain, a 12 mm iron chain along the full net length; and iii) light chain, an 8 mm iron chain along the full length. The chains were attached with cable-ties every 30 cm along the nets. These configurations were evaluated for their effectiveness in maintaining net stability and supporting seaweed growth under natural water movement.

### 4.4 Seaweed monitoring

Seaweed growth was monitored throughout the farm phase for both the line and net trials until harvest in spring. Monitoring included yield measurements (fresh and dry weight), and nutrient content analysis (carbon, nitrogen and phosphorus). These data were used to quantify biomass production and nutrient uptake, forming the basis for the analysis presented in this paper.

### 4.5 Scaling to full farm size

For the environmental and economic analysis of this paper, empirical data from the two grow-out trials were scaled to represent a full-sized farm of 18.75 hectares (750 × 250 m). The scaling assumptions were based on the spatial configuration and operational characteristics of each system: a farm using the net setup would consist of 60 pipes. Each pipe is 120 m long and holds 100 m of net, yielding 6000 m of net per cultivation site. The rows of pipes are spaced 25 m apart (Fig. 1b). The farm using the line setup assumes a seaweed line density of 1000 m per hectare (18,750 m per cultivation site), reflecting the ability to place lines closer together due to reduced manoeuvring requirements for large vessels. Lines (mainlines) are spaced 10 m apart and are 150 m long (Fig. 1b). These configurations were used to estimate material use, infrastructure needs, environmental performance, and cost at a commercial scale.

## 5 LCA methodology

This attributional LCA is compliant with ISO standards 14040 and 14044 [8,9].

### 5.1 Goal and scope

The goal of this LCA is to compare the environmental performance of different nearshore *Saccharina latissima* production systems in the Danish coasts. The main objectives were to i) calculate the environmental impact of the different production systems, ii) identify the steps that contribute the most to the environmental impact, iii) detect key trade-offs between productivity and environmental impacts, and iv) calculate the economic viability of the different production systems through NPV assessment.

The main functional unit is one seaweed cultivation area per year, which corresponds to 18,75 ha. In order to reach different target groups, additional functional units were included: 1 hectare of cultivated area per year, 1 kg of fresh or dry seaweed biomass, and 1 m of pipe or mainline.

The system boundary was defined as cradle-to-farm-gate, which includes all the production stages such as hatchery, deployment, nearshore cultivation and harvesting. Life cycle stages, including distribution, use and end-of-life treatment, were excluded from the analysis.

### 5.2 Life Cycle Inventory

The foreground data was collected from two pilot farms located in Limfjorden, Denmark (Fig. 1a). The data, which contains input materials, energy, labour and biomass yields, are compiled into the various Life Cycle Inventories (LCI). To obtain this data, the inventory corresponding to the pilot test was scaled up to a full area of 18,75 ha based on the dimensions of the cultivation area and the spacing of the pipe and mainline. The hatchery stage was scaled up based on the seeded rope production capacity of a shipping container and the required total amount of seeded rope for a full cultivation site. Background data was obtained from the Ecoinvent 3.10 and AGRIBALYSE v3.1.1 databases. The production process is divided into two stages: hatchery and cultivation, which are divided into construction and operation stages. Inventories also include disaggregated material composition of several items based on the product specifications. Materials were allocated to a year's production based on their lifetime (Eq. 1).

(1) $$Material\;allocation=\frac{total\;mass\;of\;the\;material\;\left(kg\right)}{lifetime\;of\;the\;material\;\left(years\right)}$$

### 5.3 Life Cycle Impact Assessment

Life Cycle Impact Assessment was performed using SimaPro 9.1 software and ReCiPe 2016 Midpoint (H) method [10]. For a comprehensive analysis, 18 different impact categories were included: fine particulate matter formation, fossil resource scarcity, freshwater ecotoxicity, freshwater eutrophication, global warming, human carcinogenic toxicity, human non-carcinogenic toxicity, ionising radiation, land use, marine ecotoxicity, marine eutrophication, mineral resource scarcity, ozone formation (human health), ozone formation (terrestrial ecosystems), stratospheric ozone depletion, terrestrial acidification, terrestrial ecotoxicity and water consumption. The information on cultivation area, seeded rope meters and seaweed biomass yield was used to transform the impacts per cultivation site to the other FUs.

#### 5.3.1 Emission capture calculation

To incorporate the ecosystem restorative potential of seaweed cultivation, the net environmental footprint was calculated for three impact categories: global warming potential, marine eutrophication and freshwater eutrophication. To calculate the net carbon footprint, we first calculated the CO_2_-eq. stored in the seaweed biomass by multiplying the carbon content of the biomass by 44/12. In addition, to convert the nitrogen stored in the biomass into CO_2_-eq., we used Eq. 2, where A_N_ is the amount of nitrogen assimilated in the algae biomass during growth, EF_N2O_ is the emission factor 0.005 kg N_2_O per kg N, and GWP_N2O_ is the characterisation factor to transform N_2_O into CO_2_-eq [11]. These two values were subtracted from the global warming impact to obtain the net footprint. For the net marine and freshwater eutrophication, the N and P content of the biomass, respectively, were directly subtracted from the total impacts.

(2) $$E_{N}=A_{N}\cdot EF_{N2O}\cdot \frac{44}{2\cdot 28}\cdot GWP_{N2O}$$

### 5.4 Sensitivity analysis

To evaluate the robustness of the study and the influence of key parameters on the environmental performance, four different alternative scenarios were designed.

- Lighter pipe: instead of the 315 mm thick pipe, a lighter one with a width of 110 mm was used under the assumption that seaweed does not require the same buoyancy as mussels. This scenario was simulated by reducing the weight of the pipe 8 times according to the product specifications.
- 5-year lifetime: the material required for the net and mainlines was divided by 5 to account for the extended use. It was assumed that the cleaning procedures would be the same as before for each use.
- Combined scenario: both modifications applied in the 5-year lifetime and thinner pipe scenarios were applied.
- Shipping container: the following background unit process was used instead of our values: Intermodal shipping container, 40-foot {GLO}| market for intermodal shipping container, 40-foot | Cut-off, U

## 6 Techno-Economic Assessment (TEA)

The goal of this TEA is to understand the economic viability of different nearshore *Saccharina latissima* production systems in the Danish coasts. The main objective was to: i) calculate the net present value (NPV), ii) identify the steps that contribute the most to the construction and operational expenditure (CAPEX, OPEX), iii) identify the break-even price for the systems.

The functional unit is a full seaweed cultivation area, which corresponds to 18,75 ha, over the period of a 10-year analysis horizon and 7% discount rate. The initial capital cost accounts for infrastructure investments such as containers, anchoring materials, ropes, and seeding equipment. Operational costs encompass recurring expenses related to labour, maintenance, energy consumption, and routine monitoring throughout each cultivation cycle. In alignment with the EU Commission, this study adopted a real social discount rate of 4%, as recommended for economic appraisals under EU Cohesion Policy. Since financial parameters in this techno-economic assessment are expressed in nominal terms (i.e., incorporating expected inflation), the discount rate was adjusted accordingly. Assuming an average annual inflation rate of approximately 3%, the resulting nominal social discount rate is 7%. The revenue from biomass sales was calculated based on the simulated total harvested biomass and market price per kilogram of dry seaweed. NPV is calculated under varying end-use applications and the related market prices.

NPV was calculated following Eq. 2:

(3) $$\sum_{t=0}^{n}\frac{Benefits_{t}}{{\left(1+r\right)}^{t}}-\sum_{t=0}^{n}\frac{Costs_{t}}{{\left(1+r\right)}^{t}}$$

where, n is the period of business operation (10 years), r is the discount rate, and *Benefits* and *Costs* denote the socio-economic gains and losses of biomass (seaweed) cultivation systems in year t of the 10-year business operating period, respectively.

The working capital is constant throughout the project and returned as a revenue inflow in the final year. The annual discounted balance was calculated to get the cumulative cash flow, where the present year’s cash flow is added to the previous year’s discounted balance.

## Limitations

The analysis performed in this study is based on a pilot-scale test, 6 to 30 times smaller than the simulated full cultivation site for the longline and tube-net setups, respectively. The scale-up was done linearly based on a fixed longline and pipe density, and shipping container working capacity. The productivity of the full cultivation site was scaled similarly, neglecting potential yield losses or gains derived from shading and other conditions relevant to larger scales. Moreover, due to a lack of primary and secondary data, equipment and other inputs were modelled using proxies or by only including the main components, which increases the uncertainty of the results. Due to a lack of primary data, TEA was performed using biomass selling prices retrieved from secondary data, which leads to a substantial price range due to different end-use applications, further contributing to the uncertainty in the results.

## Ethics Statement

The authors confirm that this work has followed the ethical requirements and does not involve human subjects, animal experiments or any data collected from social media platforms.

## CRediT Author Statement

**Arrate Sainz de la Maza Larrea**: investigation, Data curation, writing – original draft, writing – review & editing, methodology, validation, formal analysis, visualisation, **Manali Chakraborty**: investigation, Data curation, writing – review & editing, methodology, validation, formal analysis, visualisation, **Teis Boderskov**: resources, writing – review & editing, validation, **Mette Møller Nielsen**: resources, writing – review & editing, validation, **Annette Bruhn**: writing – review & editing, **Marianne Thomsen**: conceptualisation, supervision, writing – review & editing, methodology, formal analysis, validation, funding.

## Data Availability

zenodoDataset from the Life Cycle Assessment and Techno-Economic Analysis of net and longline nearshore Saccharina latissima cultivation systems (Original data).

## Appendix Supplementary materials

Image, application 1

## References

[1] Chakraborty M., Sainz de la Maza Larrea A., Boderskov T., Nielsen M.M., Bruhn A., Thomsen M. (2026). Comparative sustainability assessment of net and longline nearshore cultivation systems for Saccharina latissima in Danish Waters. Algal. Res..

[2] Milhazes-Cunha H., Otero A. (2017). Valorisation of aquaculture effluents with microalgae: the integrated multi-trophic aquaculture concept. Algal. Res..

[3] Seghetta M., Tørring D., Bruhn A., Thomsen M. (2016). Bioextraction potential of seaweed in Denmark - an instrument for circular nutrient management. Sci. Total Env..

[4] Kwon H., Hawkins T.R., Zaimes G.G., Infante J., Kite-Powell H.L., Stekoll M.S., Roberson L., Zotter B., Augyte S., Rocheleau G., Sims N. (2024). Life-cycle analysis of offshore macroalgae production systems in the United States. Algal. Res..

[5] Zhang X., Boderskov T., Bruhn A., Thomsen M. (2022). Blue growth and bioextraction potentials of Danish Saccharina latissima aquaculture — a model of eco-industrial production systems mitigating marine eutrophication and climate change. Algal. Res..

[6] Collins N., Kumar Mediboyina M., Cerca M., Vance C., Murphy F. (2022). Economic and environmental sustainability analysis of seaweed farming: monetizing carbon offsets of a brown algae cultivation system in Ireland. Bioresour. Technol..

[7] Greene J.M., Gulden J., Wood G., Huesemann M., Quinn J.C. (2020). Techno-economic analysis and global warming potential of a novel offshore macroalgae biorefinery. Algal. Res..

[8] International Organization for Standardization, ISO 14040:2006 environmental management - life cycle assessment - principles and framework, 2006. www.iso.org.

[9] International Organization for Standardization, ISO 14044:2006 environmental management - life cycle assessment - requirements and guidelines management environnemental, 2006. www.iso.org.

[10] M.A.J. Huijbregts, Z.J.N. Steinmann, P.M.F. Elshout, G. Stam, F. Verones, M. Vieira, M. Zijp, A. Hollander, R. Van Zelm, ReCiPe2016: a harmonised life cycle impact assessment method at midpoint and endpoint level, commentary and discussion article (2017). 10.1007/s11367-016-1246-y.

[11] Solomon S., Qin D., Manning M., Chen Z., Marquis M., Averyt K.B., Tignor M., Miller H.L., IPCC (2007). Climate Change 2007: The Physical Science Basis. Contribution of Working Group I to the Fourth Assessment Report of the Intergovernmental Panel On Climate Change.

